# Supplementary material for: Thresholds of glycemia, insulin therapy, and risk for severe retinopathy in premature infants: A cohort study
Source: PLoS Med. 2020 Dec 11;17(12):e1003477. doi: 10.1371/journal.pmed.1003477 (PMC7732100; doi:10.1371/journal.pmed.1003477)

**S1 Fig. Sensitivity analysis. ROC curve analysis of the maximum daily coefficient of variability and of the coefficient of variability of glucose values during the first 21 days of life compared to maximum value of glycemia.** Inter-day and daily variability were less informative than maximum glycemia, with lower AUC.


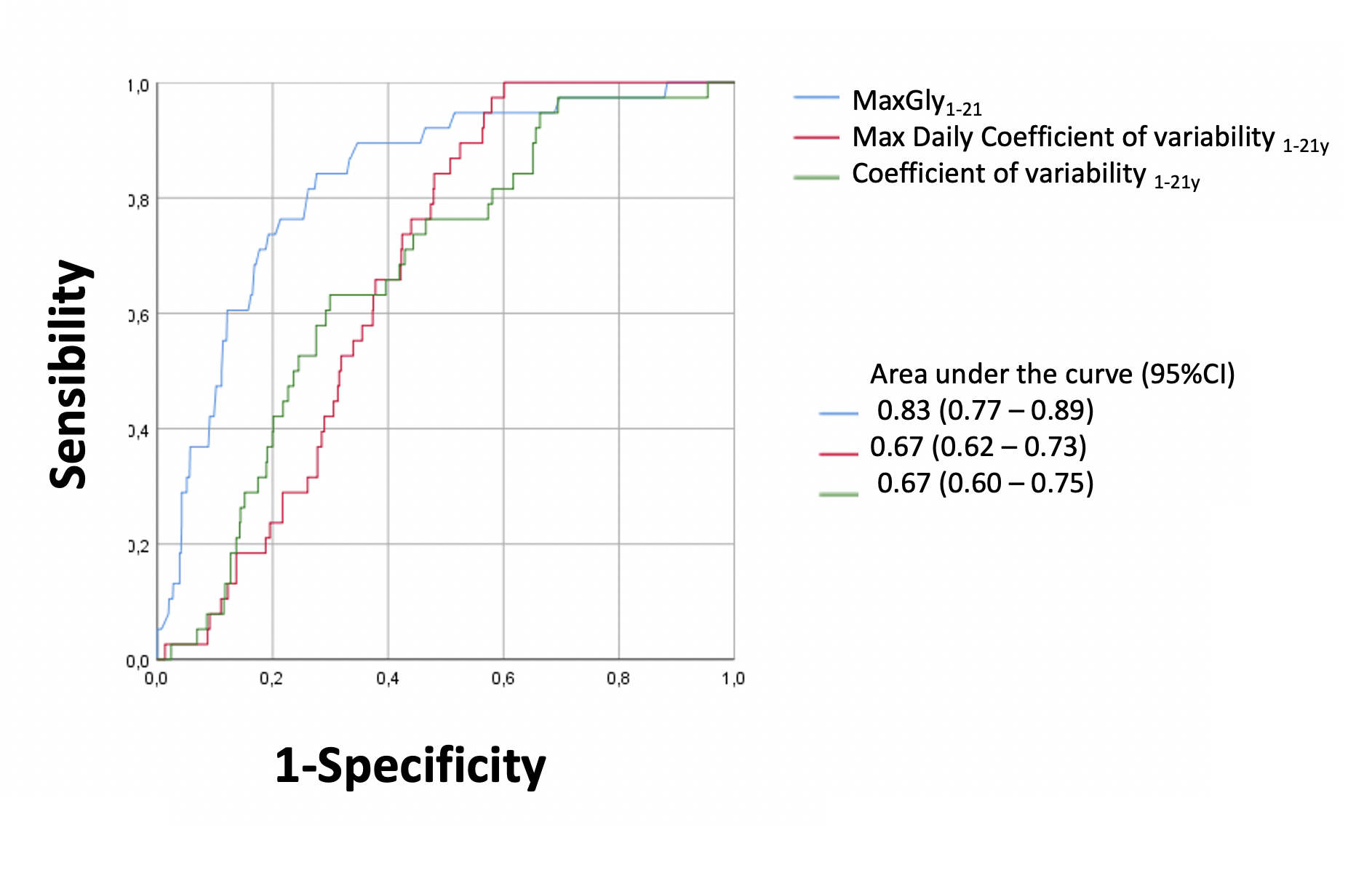

Supplement: S1 Fig — ROC curve analysis of the maximum daily coefficient of variability and of the coefficient of variability of glucose values during the first 21 days of life compared to maximum value of glycemia. (DOCX) [file pmed.1003477.s002.docx]
