# Supplementary material for: Thresholds of glycemia, insulin therapy, and risk for severe retinopathy in premature infants: A cohort study
Source: PLoS Med. 2020 Dec 11;17(12):e1003477. doi: 10.1371/journal.pmed.1003477 (PMC7732100; doi:10.1371/journal.pmed.1003477)
Supplement: S1 Table — (DOCX) [file pmed.1003477.s003.docx]

**S1 Table**

| **Analysis** | | **Primary cohort** | | | |  | **Validation cohort** | | | |
| --- | --- | --- | --- | --- | --- | --- | --- | --- | --- | --- |
|  |  | **n** | **aOR** | **95% CI** | **p** |  | **n** | **aOR** | **95% CI** | **p** |
| MaxGly_1-21_ (per mmol/l; complete cases) | |  |  |  |  |  |  |  |  |  |
|  | No adjustment | 383 | 1.2 | 1.1-1.2 | < 0.001 |  | 158 | 1.2 | 1.0-1.4 | 0.042 |
|  | Adjustment for gestational age | 383 | 1.1 | 1.1-1.2 | < 0.001 |  | 158 | 1.1 | 1.0-1.4 | 0.119 |
|  | Adjustment for birth weight Z-score | 375 | 1.2 | 1.1-1.3 | < 0.001 |  | 158 | 1.2 | 1.0-1.3 | 0.083 |
|  | Adjustment for postnatal weight gain | 375 | 1.2 | 1.1-1.3 | < 0.001 |  | 158 | 1.2 | 1.0-1.3 | 0.095 |
|  | Adjustment for duration of oxygen supplementation | 383 | 1.2 | 1.1-1.2 | < 0.001 |  | 158 | 1.1 | 1.0-1.3 | 0.100 |
|  | Adjustment for C-reactive protein | 376 | 1.2 | 1.1-1.2 | < 0.001 |  | 157 | 1.2 | 1.0-1.4 | 0.021 |
|  | Adjustment for procalcitonin | 361 | 1.2 | 1.1-1.3 | < 0.001 |  | 90 | 1.3 | 1.0-1.6 | 0.017 |
|  | Multiple adjustment including C-reactive protein ^a,c^ | 375 | 1.1 | 1.0-1.2 | 0.009 |  |  | - | - | - |
|  | Multiple adjustment including procalcitonin ^b,c^ | 361 | 1.1 | 1.1-1.2 | 0.002 |  |  | - | - | - |
| MeanMaxGly_1-21_ (per mmol/l; with multiple imputation) | |  |  |  |  |  |  |  |  |  |
|  | No adjustment | 383 | 1.5 | 1.3-1.7 | < 0.001 |  | 158 | 1.2 | 1.1-2.1 | 0.004 |
|  | Adjustment for gestational age | 383 | 1.3 | 1.1-1.6 | < 0.001 |  | 158 | 1.5 | 1.1-2.1 | 0.020 |
|  | Adjustment for birth weight Z-score | 383 | 1.5 | 1.3-1.7 | < 0.001 |  | 158 | 1.5 | 1.1-2.1 | 0.012 |
|  | Adjustment for postnatal weight gain | 383 | 1.5 | 1.3-1.7 | < 0.001 |  | 158 | 1.5 | 1.1-2.1 | 0.016 |
|  | Adjustment for duration of oxygen supplementation | 383 | 1.4 | 1.2-1.6 | < 0.001 |  | 158 | 1.5 | 1.1-2.1 | 0.020 |
|  | Adjustment for C-reactive protein | 376 | 1.5 | 1.3-1.7 | < 0.001 |  | 157 | 1.8 | 1.2-2.6 | 0.004 |
|  | Adjustment for procalcitonin | 361 | 1.5 | 1.3-1.7 | < 0.001 |  | 90 | 1.8 | 1.2-2.7 | 0.005 |
|  | Multiple adjustment including C-reactive protein ^a,c^ | 376 | 1.2 | 1.0-1.4 | 0.074 |  |  | - | - | - |
|  | Multiple adjustment including procalcitonin ^b,c^ | 361 | 1.3 | 1.0-1.5 | 0.015 |  |  | - | - | - |

**Table A. Sensitivity analysis: association between severe ROP and the maximum value of glycemia between birth and day 21 (MaxGly_1-21_) and the mean of daily maximum values of glycemia between birth and day 21 (MeanMaxGly_1-21_) in the primary and validation cohorts after adjustment for potential confounding factors in infants born at less than 28 weeks’ gestation.**

^a^ adjustment for gestational age, birth weight z-score, postnatal weight gain, duration of oxygen supplementation, and C-reactive protein; ^b^ adjustment for gestational age, birth weight z-score, postnatal weight gain, duration of oxygen supplementation, and procalcitonin; ^c^ in the validation cohort, adjustment for all potential confounders in the same model was not performed due to a too small number of cases/potential confounders ratio to estimate regression coefficients reliably.

**Table B. Sensitivity analysis: association between severe ROP and the maximum value of glycemia between birth and day 21 (MaxGly_1-21_) and the mean of daily maximum values of glycemia between birth and day 21 (MeanMaxGly_1-21_) in the primary cohort after adjustment for potential confounding factors in infants born at less than 27 weeks’ gestation**

| **Analysis** | | **Primary cohort** | | | |
| --- | --- | --- | --- | --- | --- |
|  |  | **n** | **aOR** | **95% CI** | **p** |
| MaxGly_1-21_ (per mmol/l; complete cases) | |  |  |  |  |
|  | No adjustment | 211 | 1.07 | 1.02-1.12 | 0.006 |
|  | Adjustment for gestational age | 211 | 1.06 | 1.01-1.12 | 0.026 |
|  | Adjustment for birth weight Z-score | 207 | 1.07 | 1.02-1.13 | 0.006 |
|  | Adjustment for postnatal weight gain | 207 | 1.08 | 1.02-1.13 | 0.005 |
|  | Adjustment for duration of oxygen supplementation | 211 | 1.11 | 1.02-1.19 | 0.010 |
|  | Adjustment for C-reactive protein | 206 | 1.07 | 1.02-1.12 | 0.009 |
|  | Adjustment for procalcitonin | 199 | 1.08 | 1.03-1.13 | 0.003 |
|  | Multiple adjustment including C-reactive protein ^a^ | 202 | 1.08 | 0.99-1.18 | 0.082 |
|  | Multiple adjustment including procalcitonin ^b^ | 197 | 1.11 | 1.02 – 1.22 | 0.016 |
| MeanMaxGly_1-21_ (per mmol/l; with multiple imputation) | |  |  |  |  |
|  | No adjustment | 211 | 1.32 | 1.14-1.54 | < 0.001 |
|  | Adjustment for gestational age | 211 | 1.27 | 1.08-1.49 | 0.004 |
|  | Adjustment for birth weight Z-score | 207 | 1.36 | 1.16-1.59 | < 0.001 |
|  | Adjustment for postnatal weight gain | 207 | 1.38 | 1.18-1.61 | < 0.001 |
|  | Adjustment for duration of oxygen supplementation | 211 | 1.21 | 1.02-1.42 | 0.025 |
|  | Adjustment for C-reactive protein | 206 | 1.32 | 1.13-1.53 | < 0.001 |
|  | Adjustment for procalcitonin | 199 | 1.34 | 1.15-1.57 | < 0.001 |
|  | Multiple adjustment including C-reactive protein ^a^ | 202 | 1.15 | 0.95-1.38 | 0.157 |
|  | Multiple adjustment including procalcitonin ^b^ | 197 | 1.20 | 0.99-1.46 | 0.058 |

^a^ adjustment for gestational age, birth weight z-score, postnatal weight gain, duration of oxygen supplementation, and C-reactive protein; ^b^ adjustment for gestational age, birth weight z-score, postnatal weight gain, duration of oxygen supplementation, and procalcitonin
