# Supplementary material for: Thresholds of glycemia, insulin therapy, and risk for severe retinopathy in premature infants: A cohort study
Source: PLoS Med. 2020 Dec 11;17(12):e1003477. doi: 10.1371/journal.pmed.1003477 (PMC7732100; doi:10.1371/journal.pmed.1003477)
Supplement: S2 Table — (DOCX) [file pmed.1003477.s004.docx]

**S2 Table**

**Table A. Sensitivity analysis: specificity and sensitivity of optimal cut-off values of duration of exposure above different glycemic thresholds between 7 and 13 mmol/L in the primary and validation cohorts in infants born at less than 28 weeks’ gestation.**

Among the 225 infants < 28 weeks in the primary cohort with a severe exposure to hyperglycemia (i.e. with at least one exposure above one of the optimal cut-offs), 33 (14.7%) developed a severe retinopathy, compared to only 4 (2.5%) of the 158 infants without severe exposure (p<0.001).

| **Optimal cut-off values of duration of exposure above glycemic thresholds as determined in the primary cohort** | **Primary cohort** | | | | **Validation cohort** | | | |
| --- | --- | --- | --- | --- | --- | --- | --- | --- |
|  | **Infants**  **with severe ROP**  **n=37** | **Infants without severe ROP**  **n=346** | **Specificity (95%CI)** | **Sensitivity (95%CI)** | **Infants with severe ROP**  **n=5** | **Infants without severe ROP**  **n=153** | **Specificity (95%CI)** | **Sensitivity (95%CI)** |
| 1. More than 9 days with a daily maximum above 7 mmol/L | 33 (89.2) | 158 (45.7) | 0.54 (0.50-0.59) | 0.89 (0.78-0.95) | 4 (80.4) | 64 (41.8) | 0.58 (0.52-0.65) | 0.80 (0.44-0.96) |
| 1. More than 6 days with a daily maximum above 8 mmol/L | 33 (89.2) | 164 (47.4) | 0.53 (0.64-0.57) | 0.89 (0.78-0.95) | 4 (80.4) | 61 (39.9) | 0.60 (0.54-0.66) | 0.80 (0.44-0.96) |
| 1. More than 5 days with a daily maximum above 9 mmol/L | 32 (86.5) | 143 (41.3) | 0.59 (0.72-0.78) | 0.86 (0.75-0.93 | 4 (80.4) | 56 (36.6) | 0.63 (0.57-0.70) | 0.80 (0.44-0.96) |
| 1. More than 3 days with a daily maximum above 10 mmol/L | 32 (86.5) | 146 (42.2) | 0.58 (053-0.62) | 0.86 (0.75-0.93) | 4 (80.4) | 58 (37.9) | 0.62 (0.55-0.68) | 0.80 (0.44-0.96) |
| 1. More than 2 days with a daily maximum above 11 mmol/L | 31 (83.8) | 147 (42.5) | 0.58 (053-0.62) | 0.84 (0.72-0.91) | 3 (60.0) | 58 (37.9) | 0.62 (0.55-0.68) | 0.60 (0.27-0.86) |
| 1. More than 2 days with a daily maximum above 12 mmol/L | 29 (78.4) | 115 (33.2) | 0.67 (0.62-0.71) | 0.78 (0.66-0.87) | 3 (60.0) | 42 (27.5) | 0.73 (0.66-0.78) | 0.60 (0.27-0.86) |
| 1. More than 1 day with a daily maximum above 13 mmol/L | 30 (81.1) | 119 (34.4) | 0.66 (0.61-0.70) | 0.81 (0.69-0.89) | 3 (60.0) | 46 (30.1) | 0.70 (0.64-0.76) | 0.60 (0.27-0.86) |
| Severe hyperglycemia: (a) or (b) or (c) or (d) or (e) or (f) or (g) | 33 (89.2) | 192 (55.5) | 0.55 (0.51-0.60) | 0.89 (0.78-0.95) | 4 (80.4) | 77 (50.3) | 0.50 (0.43-0.56) | 0.80 (0.44-0.96) |

**Table B. Sensitivity analysis: specificity and sensitivity of optimal cut-off values of duration of exposure above different glycemic thresholds between 7 and 13 mmol/L in the primary cohort in infants born at less than 27 weeks’ gestation.**

Among the 155 infants < 27 weeks in the primary cohort with a severe exposure to hyperglycemia (i.e. with at least one exposure above one of the optimal cut-offs), 29 (18.7%) developed a severe retinopathy, compared to only 4 (7.1%) of the 56 infants without severe exposure (p=0.041).

| **Optimal cut-off values of duration of exposure above glycemic thresholds as determined in the primary cohort** | **Primary cohort** | | | |
| --- | --- | --- | --- | --- |
|  | **Infants**  **with severe ROP**  **n=33** | **Infants without severe ROP**  **n=178** | **Specificity (95%CI)** | **Sensitivity (95%CI)** |
| 1. More than 9 days with a daily maximum above 7 mmol/L | 29 (87.9) | 107 (60.1) | 0.40 (0.33-0.47) | 0.88 (0.73-0.95) |
| 1. More than 6 days with a daily maximum above 8 mmol/L | 29 (87.9) | 108 (60.7) | 0.39 (0.33-0.47) | 0.88 (0.73-0.95) |
| 1. More than 5 days with a daily maximum above 9 mmol/L | 28 (84.8) | 95 (53.4) | 0.47 (0.39-0.54) | 0.85 (0.69-0.93) |
| 1. More than 3 days with a daily maximum above 10 mmol/L | 28 (84.8) | 97 (54.5) | 0.46 (0.39-0.53) | 0.85 (0.69-0.93) |
| 1. More than 2 days with a daily maximum above 11 mmol/L | 27 (81.8) | 95 (53.4) | 0.47 (0.39-0.54) | 0.82 (0.66-0.92) |
| 1. More than 2 days with a daily maximum above 12 mmol/L | 25 (75.8) | 74 (41.6) | 0.58 (0.51-0.65) | 0.76 (0.59-0.87) |
| 1. More than 1 day with a daily maximum above 13 mmol/L | 29 (87.9) | 116 (65.2) | 0.35 (0.14-0.89) | 0.88 (0.73-0.95) |
| Severe hyperglycemia: (a) or (b) or (c) or (d) or (e) or (f) or (g) | 29 (87.9) | 126 (70.8) | 0.29 (0.23-0.36) | 0.88 (0.73-0.95) |
