# Supplementary material for: Thresholds of glycemia, insulin therapy, and risk for severe retinopathy in premature infants: A cohort study
Source: PLoS Med. 2020 Dec 11;17(12):e1003477. doi: 10.1371/journal.pmed.1003477 (PMC7732100; doi:10.1371/journal.pmed.1003477)
Supplement: S3 Table — (DOCX) [file pmed.1003477.s005.docx]

**S3 Table. Sensitivity analysis: association between the maximum value of glycemia between birth and day 21 (MaxGly_1-21_) and a composite outcome of severe ROP or death in the primary cohort before and after adjustment for potential confounding factors.**

| **Risk for death or severe ROP** | **n** | **aOR (95%CI)** | **p** |
| --- | --- | --- | --- |
| **MaxGly_1-21_ (per mmol/l; complete cases)** |  |  |  |
| No adjustment | 1101 | 1.11 (1.08 - 1.13) | < 0.001 |
| Adjustment for gestational age | 1101 | 1.05 (1.03 - 1.07) | < 0.001 |
| Adjustment for birth weight Z-score | 1062 | 1.10 (1.08 - 1.13) | < 0.001 |
| Adjustment for postnatal weight gain | 1062 | 1.11 (1.08-1.13) | < 0.001 |
| Adjustment for duration of oxygen supplementation | 1101 | 1.10 (1.07-1.12) | < 0.001 |
| Adjustment for C-reactive protein | 1037 | 1.13 (1.10-1.16) | < 0.001 |
| Adjustment for procalcitonin | 939 | 1.16 (1.13-1.20) | < 0.001 |
| Multiple adjustment including C-reactive protein ^a^ | 1003 | 1.07 (1.04-1.10) | < 0.001 |
| Multiple adjustment including procalcitonin ^b^ | 914 | 1.11 (1.07-1.15) | < 0.001 |

^a^ adjustment for gestational age, birth weight z-score, postnatal weight gain, duration of oxygen supplementation, and C-reactive protein

^b^ adjustment for gestational age, birth weight z-score, postnatal weight gain, duration of oxygen supplementation, and procalcitonin
