# Supplementary material for: Thresholds of glycemia, insulin therapy, and risk for severe retinopathy in premature infants: A cohort study
Source: PLoS Med. 2020 Dec 11;17(12):e1003477. doi: 10.1371/journal.pmed.1003477 (PMC7732100; doi:10.1371/journal.pmed.1003477)
Supplement: S4 Table — (DOCX) [file pmed.1003477.s006.docx]

**S4 Table. Characteristics of infants according to exposure to insulin therapy in the EPIPAGE-2 cohort (N=1441).**

We applied a propensity score weighting in the EPIPAGE-2 cohort to control for observed confounding factors that might influence both group assignment, i.e. exposed (n=410, 26.7%, weighted by recruitment period) and non-exposed (n=1031) to insulin therapy. The propensity score was defined as the infants’ probability of having insulin therapy based on their individual observed covariates. Probability was estimated using a logistic regression model with insulin therapy as the dependent variable in relation to the following baseline maternal and infant characteristics: gestational age, sex, birth weight percentile, multiple birth, spontaneous preterm birth, main pregnancy complications, surfactant therapy, duration of oxygen exposure between birth and day 28, digestive state at day 7, caloric intake at day 7, and highest glycemia during the first week.

|  | **Overall Cohort ^a^** | | | |  | **Overall Cohort with Multiple Imputation ^a^** | | | | | **IPTW Cohort with Multiple Imputation^c^** | | | |
| --- | --- | --- | --- | --- | --- | --- | --- | --- | --- | --- | --- | --- | --- | --- |
|  | **Non-exposed** | | **Exposed** | |  | **Non-exposed** | | **Exposed** | | **SD, %^b^** | **Non-exposed** | **Exposed** | **SD, %^b^** | |
|  | No (%) | | No (%) | |  | Average No (%) | | Average No (%) | |  | (%) | (%) |  | |
| **Gestational age (weeks),** mean (95%CI) | 27.6 (27.5;27.7) | | 26.6 (26.5-26.7) | |  | 27.6 (27.5;27.7) | | 26.6 (26.5-26.7) | | 67.4 | 27.3 (27.1-27.4) | 27.2 (26.9-27.6) | 1.5 | |
| 24 | 22 | (1.7) | 31 | (6.6) |  | 22 | (1.7) | 31 | (6.6) | 24.7 | (3.4) | (3.6) | 0.7 | |
| 25 | 72 | (5.5) | 92 | (19.5) |  | 72 | (5.5) | 92 | (19.5) | 43.1 | (11.6) | (12.1) | 1.4 | |
| 26 | 164 | (12.6) | 107 | (22.7) |  | 164 | (12.6) | 107 | (22.7) | 26.5 | (19.2) | (17.5) | 4.5 | |
| 27 | 197 | (20.4) | 79 | (22.5) |  | 197 | (20.4) | 79 | (22.5) | 5.1 | (18.3) | (18.8) | 1.3 | |
| 28 | 277 | (28.7) | 55 | (15.7) |  | 277 | (28.7) | 55 | (15.7) | 31.8 | (23.4) | (27.2) | 8.8 | |
| 29 | 299 | (31.0) | 46 | (13.1) |  | 299 | (31.0) | 46 | (13.1) | 44.2 | (23.9) | (20.8) | 7.6 | |
| **Category of preterm birth** |  |  |  |  |  |  |  |  |  |  |  |  |  | |
| Spontaneous | 551 | (53.8) | 224 | (54.0) |  | 560 | (53.1) | 229 | (53.5) | 0.7 | (54.0) | (57.8) | 7.6 | |
| Induced | 451 | (46.2) | 174 | (46.0) |  | 471 | (46.9) | 181 | (46.5) | 0.7 | (46.0) | (42.2) | 7.6 | |
| *No information* | 29 |  | 12 |  |  |  |  |  |  |  |  |  |  | |
| **Cause of preterm birth** |  |  |  |  |  |  |  |  |  |  |  |  |  | |
| Premature labour | 438 | (41.9) | 161 | (37.5) |  | 438 | (41.9) | 161 | (37.5) | 8.9 | (40.9) | (42.7) | 3.6 | |
| Premature rupture of membranes | 272 | (25.8) | 93 | (21.7) |  | 272 | (25.8) | 93 | (21.7) | 9.5 | (24.8) | (21.7) | 7.3 | |
| Hypertensive disorder or placental  abruption | 201 | (20.3) | 88 | (23.0) |  | 201 | (20.3) | 88 | (23.0) | 6.6 | (21.2) | (20.8) | 0.9 | |
| Isolated foetal growth restriction | 47 | (4.8) | 22 | (6.2) |  | 47 | (4.8) | 22 | (6.2) | 5.9 | (4.4) | (6.2) | 8.0 | |
| Other/Undefined | 73 | (7.2) | 46 | (11.5) |  | 73 | (7.2) | 46 | (11.5) | 14.8 | (8.7) | (8.6) | 0.4 | |
| **Multiple birth** | 318 | (31.2) | 157 | (37.9) |  | 318 | (31.2) | 157 | (37.9) | 14.1 | (33.1) | (36.9) | 8.0 | |
| **Male sex** | 538 | (52.4) | 217 | (53.0) |  | 538 | (52.4) | 217 | (53.0) | 1.2 | (52.6) | (59.0) | 13.0 | |
| **Birth weight Z-score,** mean (95%CI) | 0.01  (-0.05;0.07) | | -0.48  (-0.58;-0.38) | |  | 0.01  (-0.05;0.07) | | -0.48  (-0.58;-0.38) | | 44.6 | -0.15  (-0.25;-0.06) | -0.25  (-0.46;-0.04) | 9.2 | |
| **Surfactant therapy** |  |  |  |  |  |  |  |  |  |  |  |  |  | |
| None | 193 | (20.0) | 32 | (9.0) |  | 196 | (20.0) | 33 | (9.0) | 31.8 | (16.0) | (11.8) | 12.2 | |
| 1 dose | 613 | (59.8) | 243 | (61.4) |  | 622 | (59.8) | 252 | (61.1) | 2.6 | (59.7) | (65.7) | 12.5 | |
| >2 doses | 211 | (20.2) | 119 | (29.6) |  | 214 | (20.2) | 125 | (29.9) | 22.6 | (24.2) | (22.5) | 4.2 | |
| *No information* | 14 |  | 16 |  |  |  |  |  |  |  |  |  |  | |
|  |  |  |  |  |  |  |  |  |  |  |  |  |  | |
|  | | | | | | | | | | | | | | |
| **S4 Table (continued).** | | | | | | | | | | | | | | |
|  | **Overall Cohort ^a^** | | | |  | **Overall Cohort with Multiple Imputation ^a^** | | | | | **IPTW Cohort with Multiple Imputation^c^** | | | |
|  | **Non-exposed** | | **Exposed** | |  | **Non-exposed** | | **Exposed** | | **SD, %^b^** | **Non-exposed** | **Exposed** | **SD, %^b^** | |
|  | No (%) | | No (%) | |  | Average No (%) | | Average No (%) | |  | (%) | (%) |  | |
| **Caloric intake at day 7 (kcal/kg/day)** |  |  |  |  |  |  |  |  |  |  |  |  |  | |
| 1^st^ quartile | 229 | (23.7) | 93 | (24.2) |  | 253 | (23.7) | 101 | (24.2) | 1.2 | (25.2) | (29.6) | 10.0 | |
| 2^nd^ quartile | 214 | (22.9) | 111 | (29.1) |  | 238 | (23.0) | 118 | (28.6) | 12.8 | (25.5) | (25.9) | 1.0 | |
| 3^rd^ quartile | 255 | (27.6) | 96 | (25.6) |  | 281 | (27.5) | 104 | (25.7) | 4.1 | (27.1) | (20.4) | 15.8 | |
| 4^th^ quartile | 235 | (25.8) | 79 | (21.1) |  | 259 | (25.8) | 87 | (21.5) | 10 | (22.2) | (24.0) | 4.3 | |
| *No information* | 98 |  | 31 |  |  |  |  |  |  |  |  |  |  | |
| **Maximal glycemia in the first 7 days** |  | |  | |  |  | |  | |  |  |  |  | |
| 1^st^ quartile | 307 | (34.0) | 16 | (4.2) |  | 346 | (34.3) | 17 | (4.2) | 82.5 | (24.8) | (21.6) | 7.6 | |
| 2^nd^ quartile | 304 | (32.8) | 30 | (8.1) |  | 340 | (32.8) | 32 | (8.3) | 63.7 | (25.4) | (29.4) | 8.9 | |
| 3^rd^ quartile | 221 | (23.9) | 111 | (28.1) |  | 246 | (23.8) | 116 | (28.1) | 9.9 | (24.9) | (24.2) | 1.8 | |
| 4^th^ quartile | 90 | (9.3) | 234 | (59.5) |  | 99 | (9.2) | 245 | (59.4) | 124.8 | (24.8) | (24.9) | 0.1 | |
| *No information* | 109 |  | 19 |  |  |  |  |  |  |  |  |  |  | |
| **Normal transit at day 7** | 587 | (60.7) | 172 | (45.2) |  | 619 | (60.7) | 181 | (45.2) | 31.4 | (55.8) | (53.2) | 5.2 | |
| *No information* | 54 |  | 20 |  |  |  |  |  |  |  |  |  |  | |
| **Duration of oxygen therapy (in days) between day 0 and day 28,**  mean (95%CI) | 15.3 (14.5;16.0) | | 20.9 (19.8;21.9) | |  | 15.2 (14.5-15.9) | | 20.7 (19.7-21.7) | | 45.7 | 17.1 (16.2-17.9) | 16.9 (13.7-20) | 2.0 | |
| *No information* | *23* |  | *3* |  |  |  |  |  |  |  |  |  |  | |
| Abbreviations: IPTW=Inverse Probability Weighting by Propensity Score; SD=Standardized Difference. | | | | | | | | | | | | | |  |
| ^a^ To take into account any differences in the sampling process between children included at 24–26 weeks and 27–29 weeks, results were weighted by recruitment period. | | | | | | | | | | | | | |  |
| ^b^ Standardized difference is the mean difference divided by the pooled standard deviation, expressed as a percentage. | | | | | | | | | | | | | |  |
| ^c^ Percentage are weighted by the inverse of the propensity score. | | | | | | | | | | | | | |  |
