# Supplementary material for: Thresholds of glycemia, insulin therapy, and risk for severe retinopathy in premature infants: A cohort study
Source: PLoS Med. 2020 Dec 11;17(12):e1003477. doi: 10.1371/journal.pmed.1003477 (PMC7732100; doi:10.1371/journal.pmed.1003477)
Supplement: S5 Table — (DOCX) [file pmed.1003477.s007.docx]

**S5 Table. Insulin therapy as a risk factor for severe ROP.**

Results are based on multiple imputations**.** In the main analysis, only missing data for the propensity score variables were imputed; exposure to insulin treatment and ROP status were not; 1441 babies with data on insulin and ROP were included in this analysis. The number of missing values for each variable included in the propensity score as well as data in the complete case dataset (observed values) and the imputed datasets are reported in S3 Table.

In sensitivity analyses, we also imputed exposure to insulin treatment (81 missing data, 5%) and ROP status (273 missing data, 15%); a total of 1795 infant were included.

| **Severe ROP** | No | **Non-exposed** | | **Exposed** | | **OR (95%CI)^a^** | **p-value** |
| --- | --- | --- | --- | --- | --- | --- | --- |
| ***Main analysis*** |  |  |  |  |  |  |  |
| **IPTW cohort^b^** |  |  |  |  |  |  |  |
| --- Adjusted^c^ | 1441 | 20/1031 | (3.7) | 19/410 | (2.0) | 0.40 (0.13; 1.24) | 0.106 |
|  |  |  |  |  |  |  |  |
| ***Sensitivity analyses*** |  |  |  |  |  |  |  |
| **Overall cohort** |  |  |  |  |  |  |  |
| --- Unadjusted ^d^ | 1441 | 20/1031 | (1.7) | 19/410 | (4.1) | 2.51 (1.13; 5.58) | 0.024 |
| --- Adjusted^e^ |  |  |  |  |  | 0.66 (0.28; 1.54) | 0.333 |
|  |  |  |  |  |  |  |  |
| **IPTW cohort^b^** |  |  |  |  |  |  |  |
| --- Unadjusted | 1441 | 20/1031 | (3.7) | 19/410 | (2.0) | 0.52 (0.20; 1.34) | 0.177 |
| **IPTW cohort^b, f^** |  |  |  |  |  |  |  |
| --- Adjusted^c^ | 1795 | 23/1293 | (3.6) | 23/502 | (2.2) | 0.49 (0.15-1.65) | 0.251 |
|  |  |  |  |  |  |  |  |
| Abbreviations: IPTW=Inverse Probability Treatment Weighting by Propensity Score. | | | | | | | |
| ^a^ Generalized Estimating Equations (GEE) were used to take into account NICU effects. | | | | | | | |
| ^b^ Results are weighted by the inverse of the propensity score. | | | | | | | |
| ^c^ Adjusted for the duration of oxygen therapy. | | | | | | | |
| ^d^ To take into account any differences in the sampling process between children born at 24–26 weeks’ and 27–29 weeks’ gestation , results were weighted by recruitment period. | | | | | | | |
| ^e^ Adjusted for gestational age, birth weight percentile and duration of oxygen therapy. | | | | | | | |
| ^f^ Data regarding exposure to insulin treatment and ROP status were imputed. | | | | | | | |
