# Supplementary material for: Thresholds of glycemia, insulin therapy, and risk for severe retinopathy in premature infants: A cohort study
Source: PLoS Med. 2020 Dec 11;17(12):e1003477. doi: 10.1371/journal.pmed.1003477 (PMC7732100; doi:10.1371/journal.pmed.1003477)
Supplement: S1 Text — (DOCX) [file pmed.1003477.s008.docx]

***S1 Text. Methods: preplanned analysis***

*The preplanned analysis was hypothesis-driven based on findings from rodent models of neonatal hyperglycemia-induced retinopathy published by the 1^st^ author (see reference 5 in the manuscript), and did not differ from the final analysis other than performing sensitivity analyses, and using an instrumental variable to clarify the association between insulin treatment and severe ROP, which were undertaken following peer review comments.*

Preplanned analysis (as of Dec. 20, 2016)

**Rationale:**

Several studies in recent years suggest an association between hyperglycemia (and/or insulin therapy) and an increased risk of ROP. However, these studies have important methodological weaknesses and do not rule out that hyperglycemia is only a marker of neonatal severity. Besides, experimental data in newborn rats (which retinal development is similar to that of a 26 WG preterm infant) show that neonatal hyperglycemia leads to retinal vascular degeneration as observed in the first phase of ROP.

**Questions:**

1. Does exposure to hyperglycemia during the first weeks of life (= during the 1st phase of ROP) increases the risk of ROP requiring laser treatment / ROP stage 3 or 4, when taking into account multiple confounding factors such as postnatal growth, inflammation and O2-exposure?

2. What is the effect of insulin therapy of the risk of severe ROP?

Of note, there are no randomized trials that answer the question "Does improved control of hyperglycemia limit the risk of ROP?” Such a trial would require extremely large numbers of patients, given the incidence of ROP.

**Preplanned analyses**

**1. Exposure to hyperglycemia and risk of severe ROP: LIFT cohort**

Population: < 30 WG

Endpoint: ROP stage 3 or 4

1.1. Blind review of all the retinographs in the LIFT cohort to ascertain ROP classification

1.2. Analysis of the association between severe ROP and different markers of hyperglycemia in the LIFT cohort using ROC curves:

- mean of daily maximum blood glucose levels during the first 21 days

- mean of daily maximum – minimum glycemia (delta) during the first 21 days

- maximum blood glucose value observed during the first 21 days

Comparison of the AUC;

Identification of the number of days of exposure to different levels of hyperglycemia associated with an increased risk of severe ROP.

1.3. Logistic regression models with maximum glycemia and mean daily maximum blood glucose level (introduced in the models as a continuous variable) as explanatory variables

Potential confounders to assess:

- Sex

- Gestational age

- Growth during hospitalization: delta (z-score of weight at discharge - birth weight z-score)

- Duration of oxygen therapy

- Duration of mechanical ventilation

- Inflammation/sepsis: CRP, PCT

- Number of biological examinations during the first 21 days (proxi for severity)

- Breastfeeding

- Birth weight z-score

- EPO treatment

- Pre- and post-natal corticosteroid therapy

1.4. Validation of the thresholds identified and the regression analyses in an independent monocentric cohort with similar prospective collection of clinical and biological data.

**2. Effect of insulin therapy: EPIPAGE2 cohort using a propensity score approach**

National database of more than 60 NICUs with wide variation in practices (hypothesis of a too strong link between insulin therapy and disease severity in the monocentric cohorts).

Population: < 30 WG

Endpoint: ROP stage 3 or 4

Minimum set of variables to include in the propensity score "probability of receiving insulin":

- Gestational age

- Z-score birth weight

- Maximum blood glucose level within 7 days

- Calories at D7

Potential confounders to assess:

- Delta z-score weight during hospitalization (discharge weight - birth weight)

- Insulin use

- Severe BPD

- Duration of ventilation (O2 exposure)

- NEC

- Infection

- Breastfeeding
