# Supplementary material for: Thresholds of glycemia, insulin therapy, and risk for severe retinopathy in premature infants: A cohort study
Source: PLoS Med. 2020 Dec 11;17(12):e1003477. doi: 10.1371/journal.pmed.1003477 (PMC7732100; doi:10.1371/journal.pmed.1003477)
Supplement: S2 Text — (DOCX) [file pmed.1003477.s009.docx]

**S2 Text. Methods: Imputation of missing blood glucose values.**

To calculate MeanMaxGly_1-21,_ missing blood glucose values were imputed once per day using a linear regression model; imputation model variables included glycemic data from the days before and after periods without glucose measurements, gestational age and birth weight z-score. We generated fifty independent imputed datasets. Because missing data regarding glycemia probably did not occur at random (in NICUs, blood glucose testing is not usually done in stable patients after parenteral nutrition has been discontinued because the risk of dysglycemia becomes very low), we performed sensitivity analyses with imputation of missing glucose data based on different plausible scenarios following van Buuren’s approach [27] :

1) imputation based on the best linear unbiased predictor calculated from a linear mixed-effects model adjusted on gestational age to account for the non-independence of observations within individuals, to take into account individual predictable trends in glucose concentrations ;

2) imputation at random between 4.0 and 6.9 mmol/l, within the normal range of glycemia, based on the hypothesis that the missing values of glycemia are expected to be those of the most stable patients in which glucose testing is usually withheld after a few days with normal values.
