# Supplementary material for: Thresholds of glycemia, insulin therapy, and risk for severe retinopathy in premature infants: A cohort study
Source: PLoS Med. 2020 Dec 11;17(12):e1003477. doi: 10.1371/journal.pmed.1003477 (PMC7732100; doi:10.1371/journal.pmed.1003477)
Supplement: S3 Text — (DOCX) [file pmed.1003477.s010.docx]

**S3 Text. Sensitivity analysis: insulin therapy as a risk factor for severe ROP - instrumental variable.**

To reduce bias from unmeasured confounding, we applied instrumental variable analyses using the two-stage residual inclusion method.

We used NICU preference regarding insulin use as the instrumental variable.

We estimated the unit effect adjusted for all baseline characteristics (variables included in the propensity score model). We used a mixed-effects logistic regression model with fixed effects for the baseline characteristics and a random unit effect. We categorized the estimated random unit effects into quartiles to define our instrumental variable. To assess its validity, we confirmed that the unit effect was correlated with insulin treatment (Table A). We also examined covariate balance across the estimated quartile of unit effect (Table B).

**Table A**. Association between the instrumental variable and exposure to insulin treatment in the overall cohort (N=1441)

| **Instrumental variable^a^** | **Exposed n/ N** | **% insulin treatment** |
| --- | --- | --- |
| Quartile 1 | 20/339 | 5.3 |
| Quartile 2 | 71/378 | 16.9 |
| Quartile 3 | 151/373 | 38.1 |
| Quartile 4 | 168/351 | 45.5 |
| ^a^ Instrumental variable is the unit preference regarding insulin use. | | |

**Table B.** Instrumental variable and covariates

|  | **Instrumental Variable** | | | |  |
| --- | --- | --- | --- | --- | --- |
|  | **Quartile 1** | **Quartile 2** | **Quartile 3** | **Quartile 4** | **p trend** |
|  | **n=339** | **n=378** | **n=373** | **n=351** |  |
| **Gestational age (weeks),** mean (SD) | 27.2 (1.4) | 27.6 (1.3) | 27.3 (1.4) | 27.4 (1.4) | 0.611 |
| **Cause of preterm birth** |  |  |  |  |  |
| Preterm labor group | 41.1 | 43.3 | 38.5 | 39.8 | 0.192 |
| Preterm premature rupture of membranes | 27.1 | 23.2 | 24.2 | 24.7 |  |
| Hypertensive disorders or placental abruption group | 20.0 | 22.8 | 20.4 | 20.7 |  |
| Isolated fetal growth restriction | 4.2 | 3.1 | 6.4 | 7.2 |  |
| Other/not defined | 7.5 | 7.6 | 10.5 | 7.6 |  |
| **Multiple births** | 34.6 | 30.8 | 33.2 | 33.5 | 0.981 |
| **Male sex** | 53.8 | 49.7 | 50.1 | 57.0 | 0.377 |
| **Birth weight Z-score,** 95%CI | 0.12 (1.05) | 0.02 (0.99) | 0.21 (0.97) | 0.14 (1.02) | 0.256 |
| **Surfactant** |  |  |  |  | 0.190 |
| No | 10.1 | 20.6 | 21.2 | 15.4 |  |
| 1 dose | 63.7 | 59.7 | 56.9 | 60.8 |  |
| >2 doses | 26.2 | 19.8 | 22.0 | 23.8 |  |
| **Caloric intake at day 7 (kcal/kg/day), mean (SD)** | 93.0 (21.5) | 101.6 (17.9) | 106.1 (22.5) | 109.6 (17.7) | <0.001 |
| **Maximal glycemia in the first 7 days** | 9.2 (3.4) | 9.6 (4.4) | 10.5 (4.8) | 10.1 (4.5) | <0.001 |
| **Normal transit at day 7, mean (SD)** | 53.8 | 62.1 | 54.6 | 55.2 | 0.649 |
| **Duration of oxygen therapy (in days) between day 0 and day 28, mean (SD)** | 16.6 (11.8) | 17.5 (11.9) | 15.7 (11.8) | 16.9 (11.3) | 0.688 |

Instrumental variable analysis was carried out using the two-stage residual inclusion approach. In the first stage, the residual between the observed and the predicted probability of insulin treatment given the instrumental variable was computed from logistic regression. In the second stage, we analysed associations between insulin treatment and outcomes using logistic models adjusted for confounding factors and the residual of the first model as indicating unmeasured confounders.

**We obtained an adjusted OR of 0.50 [95% CI, 0.14 to 1.85] pv=0.300.**
