# Supplementary material for: Thresholds of glycemia, insulin therapy, and risk for severe retinopathy in premature infants: A cohort study
Source: PLoS Med. 2020 Dec 11;17(12):e1003477. doi: 10.1371/journal.pmed.1003477 (PMC7732100; doi:10.1371/journal.pmed.1003477)
Supplement: Attachment — Submitted filename: Kermorvant_et_al_Response_to_reviewers.docx [file pmed.1003477.s011.docx]

**Kermorvant-Duchemin et al.**

**Thresholds of glycemia, insulin therapy and risk for severe retinopathy in premature infants: A multiple cohort study.**

PMEDICINE-D-19-03700

Response to reviewers and editors

**Requests from the editors:**

*At this stage, we ask that you include a short, non-technical Author Summary of your research to make findings accessible to a wide audience that includes both scientists and non-scientists. The Author Summary should immediately follow the Abstract in your revised manuscript. This text is subject to editorial change and should be distinct from the scientific abstract. Please see our author guidelines for more information: https://clicktime.symantec.com/3Fwk3rtqFSySEw1Uimv8e746H2?u=https%3A%2F%2Fjournals.plos.org%2Fplosmedicine%2Fs%2Frevising-your-manuscript%23loc-author-summary

*Answer:*

*We added an Author Summary has been in the revised manuscript page 5 and 6.*

*Please clarify in the paper if the analytical approach reported here corresponds to one laid out in a prospective protocol or analysis plan? Please state this (either way) early in the Methods section.

a) If a prospective analysis plan (from your funding proposal, IRB or other ethics committee submission, study protocol, or other planning document written before analyzing the data) was used in designing the study, please include the relevant prospectively written document with your revised manuscript as a Supporting Information file to be published alongside your study, and cite it in the Methods section. A legend for this file should be included at the end of your manuscript. 
b) If no such document exists, please make sure that the Methods section transparently describes when analyses were planned, and when/why any data-driven changes to analyses took place.  
c) In either case, changes in the analysis-- including those made in response to peer review comments-- should be identified as such in the Methods section of the paper, with rationale.

*Answer:*

*No prospective study protocol was written before analyzing the data.*

*The preplanned analysis was hypothesis-driven based on findings from rodent models of neonatal hyperglycemia-induced retinopathy published by the 1^st^ author (see reference 5), and did not differ from the final analysis other than performing sensitivity analyses, and using an instrumental variable to clarify the association between insulin treatment and severe ROP, which were undertaken following peer review comments.*

*This was clarified in the Methods section, in the paragraph Statistical analysis, page 10 of the revised manuscript.*

*We noted a couple of areas particularly in the Discussion (but may also be in other parts of the paper) where the reporting immediately goes to assuming that the effects observed in this analysis represent causal effects (despite that elsewhere the authors acknowledge the possibility of residual confounding. For example in the discussion (1st sentence) - "we found...that high glucose exposure in the first 3 weeks of life **increased** the risk of severe ROP"; (final para of discussion) - "our study identified thresholds levels of combined duration and blood glucose concentration that increase the risk of severe ROP significantly...". Both might be better stated as "associated with increased risk" for example.

*Answer:*

*We acknowledge that the formulation overstated what the study may allow to conclude. The manuscript has been modified so as to lower the tone of any statement implying a causal effect. In particular, we replaced all the occurrences of “increased the risk” by "is/are associated with (an) increased risk of" in the revised manuscript.*

**Reviewer #1:**

The authors examined the impact of different levels of hyperglycemia on the risk of severe retinopathy in premature infants. The authors showed, on the basis of three independent cohorts, that high levels of glucose were an independent risk factor for developing retinopathy in premature infants. The study was conducted in a unique study sample, and conducting the study in three independent samples is a major strength. 

*Answer:*

*We thank the reviewer for this comment.*

I have a number of comments/suggestions that need to be addressed before this manuscript should be considered for publication.

1) Somewhat more details should be given about the study populations.

**How were the infants included in the study?**

*Answer:*

*In the two primary and validation cohorts, all children hospitalized consecutively in each center during the study period were included unless their parents objected thereto.*

*The EPIPAGE-2 cohort is a nationwide prospective population-based cohort with a defined period of recruitment over all regions of France, except one (Ancel et al., BMC Pediatr. 2014;14:97).*

*We clarified this point in the Methods section, first paragraph, page 8 of the revised manuscript.*

**How was the participation rate?**

*Answer: the participation rate was 100% in the primary and the validation cohorts before considering exclusion criteria (death before 36 weeks).*

*In the EPIPAGE-2 study, participation rates were 92.6% among infants born at 22 through 26 weeks and 93.6% among those at 27 through 31 weeks (Ancel et al, JAMA pediatrics 2015).*

**Was written informed consent from the parents available?**

**It is not clear form the text whether the cohort was a routine care database (and thus retrospective) or a prospective cohort study (with inclusion etc etc).**

*Answer:*

*The primary and the validation cohorts are institutional cohorts where medical records are fully computerized and clinical and biological data prospectively collected. The EPIPAGE-2 cohort is a national population-based prospective cohort study.*

*According to French law (Law No. 2012-300 of 5 March 2012 on research involving human subjects, as amended by Ordinance No. 2016-800 of 16 June 2016 and its implementing decrees n° 2016-1537 of 16/11/2016 and n° 2017-884 of 9/05/2017), observational studies can be conducted on a subject without written consent, provided that the subject has been informed of the research and has not raised an objection thereto.*

*Written information about the use of clinical and biological data for clinical research aimed at improving care is provided to all parents whose children are included in the 2 institutional cohorts. During the study period, no parental objections were expressed.*

*In the national prospective cohort EPIPAGE-2, recruitment and data collection occurred only after families had received information and agreed to participate in the study. Parents and/or legal guardians of all infants provided oral informed consent at enrolment.*

*Some details of recruitment have been published previously and references to the papers are indicated in the text; we added other details of the study populations in the revised manuscript (page 8, Methods section).*

2) I would recommend to add AUC values to the ROC curves (Figure 2A).

*Answer: we added the AUC values and the corresponding 95% confidence intervals to each ROC curve in Figure 2A.*

3) A high proportion of the infants died before enrollment in the analyses. Did the authors considered competing risks in the analyses given that hyperglycemia and mortality could be related as well?

*Answer:*

*We agree with the reviewer that hyperglycemia and mortality could be associated. To further explore possible biases related to competing risks between mortality and ROP, we made a sensitivity analysis in the primary cohort to study the association between maximum value of glycemia (MaxGly_1-21_) and a composite outcome of death or severe ROP, before and after adjustment for potential confounders. As shown in the table below, the association between hyperglycemia and the composite outcome of death or severe ROP was lower than with ROP only.*

*We added this table in the supporting information file (S3 Table).*

| Risk for death or severe retinopathy | **n** | **aOR**^a^ (95%CI) | **P** |
| --- | --- | --- | --- |
|  |  |  |  |
| **MAIN ANALYSIS** |  |  |  |
| **MaxGly_1-21_ (per mmol/L; complete cases)** |  |  |  |
| No adjustment | 1101 | 1.11 (1.08 - 1.13) | 0.001 |
| Adjustment for gestational age ^$,§^ | 1101 | 1.05 (1.03 - 1.07) | 0.001 |
| Adjustment for birth weight Z-score ^$,§^ | 1062 | 1.10 (1.08 - 1.13) | 0.001 |
| Adjustment for postnatal weight gain ^$,§^ | 1062 | 1.11 (1.08-1.13) | 0.001 |
| Adjustment for duration of oxygen supplementation^$,§^ | 1101 | 1.10 (1.07-1.12) | 0.001 |
| Adjustment for C-reactive protein ^$^ | 1037 | 1.13 (1.10-1.16) | 0.001 |
| Adjustment for procalcitonin ^§^ | 939 | 1.16 (1.13-1.20) | 0.001 |
| Adjustment for all confounders marked with ^$,b^ | 1003 | 1.07(1.04-1.10) | 0.001 |
| Adjustment for all confounders marked with ^§,b^ | 914 | 1.11 (1.07-1.15) | 0.001 |

4) It would have been of interest not only to look at the mean of peak glucose value, but also to take into account the inter-day variability in glucose levels in relation to ROP. Finding something here would make a stronger argument to frequently measure glycemic levels.

*Answer:*

*We thank the reviewer for this interesting suggestion. We performed a sensitivity analysis to explore the association between inter-day and daily variability in glucose and severe ROP. The figure below shows the ROC curves of the maximum daily coefficient of variability and of the coefficient of variability of glucose values during the first 21 days of life compared to maximum glycemia. Inter-day and daily variability were less informative, with lower AUC. We added this figure in the Supporting information file (S1 Fig).*

5) The number of measures of glycemic levels could be related to ROP, as an indication of glycemic variability.

*Answer:*

*This is also an interesting suggestion. The number of measures of glycemic levels was correlated to the coefficient of variability of glucose values_1-21_ , but with a very low R^2^ (R^2^ = 0.02). In addition, the association between severe ROP and variability of glycemia was weak as shown above.*

6) Table 3. It would have been informative not to change both the duration and maximum value at the time, which makes the analyses difficult to care to each other.

*Answer: in table 3, the duration of exposure and glycemic level that are indicated in the first column correspond to each optimal cut-off values of duration of exposure above the corresponding glycemic level as determined in the primary cohort based on the ROC curves.*

*We made the Table 3 legend clearer by modifying its phrasing to:*

*“Specificity and sensitivity of the optimal cut-off values of duration of exposure above the corresponding blood glucose concentration (as determined in the primary cohort based on the Youden’s index) in the primary and the validation cohorts.”*

7) Page 18. Which confounding factors had most impact on the observation?

It is perhaps strange that the effect estimate becomes protective. I am not sure whether propensity scores will exclude the possibility of confounding by indication in this analysis.

Can the authors comment on this?

Did the authors perform a predefined power calculation?

Given the low use of insulin in combination with a low prevalence of ROP, it is quite likely you will easily run out of statistical power.

*Answer:*

*The main confounders were related to indication bias, as the reviewer points out. The unadjusted association showed an OR >>1 between insulin and retinopathy due to confounding by indication. After taking into account the propensity score (to reduce the indication bias), we observed a reversed, protective effect estimate with an OR of 0.40 (95%CI, 0.13 to 1.24). The omission of a confounder would tend to reduce the observed protective effect and cannot explain the reversal of the estimate. To confirm this, we performed a sensitivity analysis using an instrumental variable (using unit preference regarding insulin use as instrument), despite a limited power due to the low rate of retinopathy in this large nationwide cohort. We obtained a similar odds ratio of 0.50 (95% CI, 0.14 to 1.85). We added this sensitivity analysis* *in the Supporting Information File (S1 Data).*

*We did not performed a predefined power calculation because we used an existing prospective nationwide population-based cohort. A retrospective power analysis showed that enrollment of 1400 infants with a 30% rate of exposition can detect an OR of 0.3 with a risk alpha of 0.05 and beta of 0.20.*

**Reviewer #2:**

I confine my remarks to statistical aspects of this paper. These were generally fine but I have a couple issues to resolve before I can recommend publication.

**First, why divide the stages of ROP into two? I looked up the 5 stages and surely stage V is worse than IV and IV worse than III.  So ... I suggest leaving ROP as a 5 level variable and using ordinal logistic regression.**

*Answer:*

*Indeed, the International Classification of Retinopathy of Prematurity (ICROP) from stage 1 to stage 5 describes increasing severity of the disease.*

*Stage 1 and 2 (mild ROP) do not usually require treatment as the disease regresses in 80% of cases.*

*Progression from stage 2 to stage 3 is critical since it puts the baby at risk of retinal detachment which occurs in up to 40 to 50% of stage 3 ROP unless treatment (laser retinal ablation in most cases) is performed.*

*Stage 4 et 5 correspond to partial (stage 4) or total (stage 5) retinal detachment, leading to severe visual impairment or even blindness that can seldom be reversed despite eye surgery.*

*Stage 3 (severe ROP) corresponds to the stage where laser treatment is generally needed to prevent progression of ROP towards stage 4/5. Laser retinal ablation modifies the natural course of the disease and reduces unfavorable visual outcome (retinal detachment) from around 50 to 15% according to the main RCTs. Incidence of stage 4/5 ROP is therefore lowered by laser treatment and low numbers may preclude analyses with sufficient power in the cohorts.*

*Therefore, we think it is more relevant, from a clinical point of view, to study the association between hyperglycemia and stage 3 or more ROP instead of leaving ROP as a 5 level variable.*

**Line 208: Please define the Youden index. Not everyone will know what it is.  More importantly, you have to justify its use. Maximizing the Youden index assumes that false positive and false negative conclusions are equally bad. I don't know much about ROP or insulin treatment, but it's not, in general, the case that the two errors are equally bad. This needs justification or modification.**

*Answer:*

*We added the definition of the Youden index in the Methods section as follows:*

*“For each concentration of glycemia, we analyzed the association between exposure time and severe ROP using ROC curves, and identified the most discriminatory value of exposure time (“optimal cut-off “) as the cut-off value with the highest Youden index, a common summary measure of the ROC curve which defines the maximum potential effectiveness of a marker by optimizing both its sensitivity and sensibility”.*

*We agree that maximizing the Youden index leads to an optimal threshold (in the sense of minimizing the rate of misclassification) when the ‘cost’ of false negatives and false positives are equal, and prevalence is 0.5 (e.g. Perkins & Schisterman, American Journal of Epidemiology 2006;163:670–675). Our setting is slightly different from the classical diagnostic test evaluation, in that the prevalence of ROP would be causally linked to the duration of hyperglycemia, so that accepting a higher threshold would induce a higher incidence of ROP. But the issue of the relative cost of false negatives and false positives is indeed crucial. It is complex to elicit such costs, because they depend on the burden for patients but also on medical and society costs incurred with false positives and false negatives. For the false negatives, those costs relate to the consequences of ROP.*

*We performed a sensitivity analysis with a ratio of the cost of false negatives/cost of false positives varying from 0.5 to 4.*

| *Ratio cost of false(-)/cost false(+)* | *0.5* | *1 (Youden)* | *2* | *4* |
| --- | --- | --- | --- | --- |
| *Number of days as cut-off for 7 mmol* | *11* | *9* | *9* | *9* |
| *Number of days as cut-off for 8 mmol* | *7* | *6* | *6* | *6* |
| *Number of days as cut-off for 9 mmol* | *6* | *5* | *5* | *4* |
| *Number of days as cut-off for 10 mmol* | *4* | *3* | *3* | *3* |
| *Number of days as cut-off for 11 mmol* | *4* | *2* | *2* | *1* |
| *Number of days as cut-off for 12 mmol* | *2* | *2* | *1* | *1* |
| *Number of days as cut-off for 13 mmol* | *1* | *1* | *1* | *1* |

*In the clinical setting, we think the adapted ratio is more than 1, as undesirable as ROP is.*

*However, since differences in duration of exposure are limited when changing the ratio of the cost of false negatives/cost of false positives between 1 and 4, we suggest to maintain the primary analysis with a threshold obtained by maximizing the Youden index, a more frequently used approach.*

**Reviewer #3:**

Review of article "Thresholds of glycemia, insulin therapy and risk for severe retinopathy in premature infants: A multiple cohort study."

I have no competing interest on this subject.

Review:
1. This paper does address an important question if hyperglycemia is associated with worsening ROP, a significant important disease affecting extremely preterm infants.

*Answer:*

*We thank you the reviewer for this comment.*

2. I feel that this paper does not advance the field –

*Answer:*

*Current published evidence in the field, based mostly on small retrospective cohorts, does not rule out that hyperglycemia may only be a marker of severity of illness and not an independent risk factor for retinopathy of prematurity.*

*Based on a unique and large set of data from three independent cohorts, our results establish that hyperglycemia is an independent risk factor for retinopathy of prematurity with the most complete adjustment possible. Multiple sensitivity analyses and external validation strengthens the findings and increases generalizability of our results.*

*The most important component of the study is the determination of optimal cutoff values for combined severity of hyperglycemia and duration of exposure and the risk for severe ROP, which we termed "severe exposure". Taking into account both the duration and the level of hyperglycemia represents a truly novel approach in evaluating exposure to hyperglycemia.*

*We believe that the clinical impact of our results will be important in helping neonatologists to determine when to treat hyperglycemia in preterm infants. Our study will also help to design randomized control trials evaluating treatment strategies in hyperglycemic premature infants.*

There are specific concerns in this paper:

a. Line 41 states hyperglycemia causes tissue damage - this is not supported in literature - it may be associated but not causative.

*Answer:*

*We modified the sentence “However, it is uncertain which concentration of blood glucose could cause tissue damage, with little consensus on the cut-off level to treat hyperglycemia.” to “However, it is uncertain which concentration of blood glucose is associated with increased risk of tissue damage, with little consensus on the cut-off level to treat hyperglycemia.”*

b. The level of hyperglycemia is quite elevated - for example 63% of infants had glucose 180 mg/dL and 29% were greater than 20 mg/dL.

*Answer:*

*Severe glucose dysregulation is common in neonatal intensive care; its incidence varies between 30 and 80% depending on gestational age and the threshold used to define hyperglycemia. In the NIRTURE study evaluating early insulin treatment in premature infants < 1500g, glucose levels >8 mmol/L (150 mg/dL) occurred in 80% of infants, glucose levels >10 mmol/L (180 mg/dL) occurred in 57% of infants, glucose levels >15 mmol/L (270 mg/dL) in 23% of infants, and 9% of infants had at least 1 glucose reading >20 mmol/L (360 mg/dL) (Beardsall et al., J Pediatr 2010).*

*These figures are in the same order of magnitude than those we observed, since MaxGly_1-21_ was higher than 8 mmol/L in 79.5% of the infants, >10 mmol/l in 62.6%, >15 mmol/L in 29.0%, and >20 mmol/L in 12.9% in the primary cohort.*

c. Line 73-5 contrary to what the authors state - the incidence of severe ROP has decreased over the time of this study due to improved control of supplemental oxygen delivery.

*Answer:*

*In the study by Stoll et al. examining the outcome of 34636 infants born 22 to 28 weeks’ gestation, ROP of stage 3 or higher increased from 13% of infants (124 of 941) in 1993 to 19% (262 of 1385) in 2003, then decreased to 11% of infants (160 of 1509) by 2012 (adjusted RR (95% CI), change per year 1993-2003: 1.02 (1.01-1.03) 2004-2012: 0.94 (0.93-0.95)), representing a limited change between 1993 and 2012. In particular, no change was observed for infants born at 22 through 24 weeks. During that period, survival increased, therefore the raw number of infants surviving with severe ROP also increased.*

*Other papers described a recent increase in incidence of ROP. Manley et al. reported that changing oxygen saturation target ranges for extremely preterm infants from 88%-92% to 91%-95% in their center had been associated with increased rates and severity of ROP (Manley BJ, et al., J Pediatr 2016;168:242-4). In an Australian prospective longitudinal cohort study that was published very recently, retinopathy of prematurity requiring treatment increased from 4.0% (1991–1992) to 10.0% (2016–2017) (Cheong JLY, et al. BMJ Open 2020;10:e037507).*

*Thus, changes in incidence of ROP are difficult to describe concisely and the phrasing*

*“Retinopathy of prematurity (ROP) is a multifactorial sight-threatening disease that remains a challenge in neonatal care, with limited change in incidence in the past 20 years, but increasing rates of survival among at risk infants” seemed prudent. However, we are disposed to change this sentence should the reviewer and the editors request it.*

d. The authors do not state the level of C reactive protein and calcitonin that is elevated - IE a surrogate for sepsis and marker of inflammation. They also don't state the timing of and the number of values obtained per infant. Elevation of these can be a late finding in sepsis.

*Answer:*

*C-reactive protein and procalcitonin were entered in the multiple regression analysis models as continuous variables, as other confounders.*

*This has been indicated in the notes below Table 2.*

*The median number of C-reactive protein measures during the first 21 days after birth was 6 (IQR [3,12]). The median age when maximal level of C-reactive protein was observed was day 3 (IQR [1,12]).*

*The median number of procalcitonin measures during the first 21 days after birth was 6 (IQR [2,8]). The median age when maximal level of procalcitonin was observed was day 9 (IQR [0,14]).*

e. Lines 190-197 are wordy and difficult to understand.

*Answer:*

*We simplified the paragraph in the main text as follows and we moved the complete details of the original paragraph in the supporting information file.*

*“We generated fifty independent imputed datasets. Because missing data regarding glycemia did probably not occur at random (in NICUs, blood glucose testing is usually withheld in stable patients after parenteral nutrition has been discontinued because the risk of dysglycemia becomes very low), we performed sensitivity analyses with imputation of missing glucose data based on different plausible scenarios following van Buuren’s approach [27] (see S1 Text in the Supporting Information for details).”*

f. In table 1, the 2 populations are not comparable in survival, change in weight, and incidence of BPD.

*Answer:*

*Differences in survival, change in weight gain and incidence of BPD are common between neonatal units and reflect differences in unit policies. Within the EPIPAGE 2 cohort, several papers have shown a large variation of practices due to large differences in unit policies (see for example Rozé JC et al, JAMA Netw Open. 2020;3(9):e2018119, Rozé JC et al, Am J Clin Nutr. 2017;106:821-830). Confirmation of the association between hyperglycemia and severe ROP in the validation cohort is in support of the independence between the observed association and unit policies.*

g. Around line 282 - It appears that most of the infants with elevated glucose had significantly elevated values as it appears that most had at least one level > 13 mmol/L (234 mg/dL).

*Answer:*

*Infants who had at least one day of exposure to glycemia > 13 mmol/L represented 23.4% of the primary cohort.*

*Most of those who developed a severe ROP had at least one day of exposure to glycemia > 13 mmol/L.*

*The paper shows an independent association between exposure to hyperglycemia and severe ROP, which can be studied using different definitions, such as maximum glycemia (MaxGly_1-21_), or exposure to a combined duration and level of glycemia. Taking into account the different thresholds of combined level and duration of hyperglycemia reflects the area under the curve and increases the sensibility of severe exposure (defined as exposure to at least one duration of exposure above the corresponding threshold of MaxGly_1-21_) to predict severe ROP.*

*Being exposed to any situation in the “danger zone” of severe exposure we identified is associated with a tenfold increased risk of severe ROP (figure 2B).*

h. This paper would be enhanced if they focused on infants with gestational age < 27 weeks and infants with gestational age > 26 weeks rarely have severe ROP.  Including the older infants in the analysis does not add much and may confound the data evaluation.

*Answer:*

*We agree with the reviewer that infants less than 27 weeks have more frequently severe ROP than infants > 26 weeks; however they are not spared from it as shown in the paper by Stoll et al. in 2015, based on data from the Neonatal Research Network in the USA. Also, current screening guidelines from many high-income countries encompass a larger group of infants than > 26 weeks (i.e., up to 31 weeks GA).*

*Gestational age was taken into account as a confounder in the regression analyses.*

*Restricting the main analysis to infants less than 27 weeks would have reduced the power of the study since these infants are much less numerous, as observed in the sensitivity analysis of the association between severe ROP and MaxGly_1-21_ and MeanMaxGly_1-21_* *in infants < 27 weeks that we added in the Supporting Information File (S1b and S2b Tables of the revised Supporting information file, following the same analysis in infants less than 28 weeks that was presented in the original Supporting information).*

3. This topic is of general interest to neonatologists, pediatricians, and pediatric ophthalmologists.

*Answer:*

*Thank you for your comment, with which we fully agree.*
