# Supplementary material for: Thresholds of glycemia, insulin therapy, and risk for severe retinopathy in premature infants: A cohort study
Source: PLoS Med. 2020 Dec 11;17(12):e1003477. doi: 10.1371/journal.pmed.1003477 (PMC7732100; doi:10.1371/journal.pmed.1003477)
Supplement: Attachment — Submitted filename: Kermorvant_et_al_Response_R2.docx [file pmed.1003477.s012.docx]

**Kermorvant-Duchemin et al.**

**Thresholds of glycemia, insulin therapy and risk for severe retinopathy in premature infants: A cohort study.**

PMEDICINE-D-19-03700

**Responses to requests from editors and comments from reviewers.**

1. Please address any final review comments below.

*Please see our answers below.*

2. Title: Please amend to: “Thresholds of glycemia, insulin therapy and risk for severe retinopathy in premature infants: A cohort study.”

*We amended the title as requested.*

3. Data Availability Statement: Since the data are available on request, please amend your response to the first question, which currently states “Yes - all data are fully available without restriction”. Please then also describe the reasons for why data are only available on request, eg. patient confidentiality.

*We amended our response concerning the Data Availability Statement.*

*Data from the primary and the validation cohorts*

*Despite anonymisation, the data from the primary and the validation cohorts used for the analyses contains potentially identifying patient information, in particular in the validation cohort where the small number of ROP combined with gestational age may allow patient identification. In compliance with the European General Data Protection Regulation, these data cannot be shared in a public repository.*

*Data from the EPIPAGE-2 cohort*

*According to the rules imposed by the INSERM (Institut National de la Santé et de la Recherche Médicale), sponsor of the EPIPAGE-2 study, access to the EPIPAGE-2 data is subject to authorisation by the cohort Data Access Committee, in accordance with the European General Data Protection Regulation and the new law for modernisation of the French Public Health System voted in 2016.*

*The data underlying the results presented in the study are all available from Valerie Benhammou (valerie.benhammou@inserm.fr) on request from researchers meeting the criteria for access to confidential data.*

*Data contact person:*

*Valérie Benhammou*

*INSERM U1153 - Equipe EPOPé*

*Bâtiment Recherche - Hôpital Tenon
4, rue de la Chine 75020 Paris, France*

*valerie.benhammou@inserm.fr*

4.	Abstract:

a.	Please include cohort recruitment dates, and the setting(s). Please remember to include the number of participants in the EPIPAGE-2 cohort.

*We added the above recruitment details in the abstract.*

b.	Please include summary participant demographics (age, sex).

*We added the summary participants demographics in the abstract.*

c.	Please quantify all results with p values and 95% CIs, eg. line 50.

*We added p values and 95% CIs as follows:*

*“MaxGly1-21 and MeanMaxGly1-21 were significantly associated with an increased risk of severe ROP: OR 1.21 [95% CI, 1.14-1.27], p<0.001 and OR 1.70 [95% CI, 1.48-1.94], p<0.001 respectively in the primary cohort and OR 1.17 [95% CI, 1.05-1.32], p=0.008 and OR 1.53 [95% CI, 1.20-1.95], p<0.001 respectively in the validation cohort. These associations remained significant after adjustment for confounders in both cohorts.”*

d.	Line 61: Please name the main factors that were adjusted for.

*We amended the text as follows:*

*“Adjustment for inverse propensity score and duration of oxygen therapy had a large but not significant effect on the association between insulin treatment and severe ROP (OR 0.40 [95% CI, 0.13-1.24], p=0.11).”*

*Another possibility, though longer, would be naming all the covariates taken into account in the propensity score:*

*“Adjustment for inverse propensity score (gestational age, sex, birth weight percentile, multiple birth, spontaneous preterm birth, main pregnancy complications, surfactant therapy, duration of oxygen exposure between birth and day 28, digestive state at day 7, caloric intake at day 7, and highest glycemia during the first week) and duration of oxygen therapy had a large but not significant effect on the association between insulin treatment and severe ROP (OR 0.40 [95% CI, 0.13-1.24], p=0.11).”*

e.	Line 64: Please replace “subjects” with “patients”.

*We replaced “subjects” with “patients”.*

f.	Line 67: Please rephrase as: “In this study, we observed that exposure…”

*We rephrased the sentence as requested.*

5.	Author summary:

a.	Lines 75 and 79: Please briefly define ‘hyperglycemia’ and ‘retinopathy’ to a lay reader.

*We defined hyperglycemia and retinopathy as follows:*

- *Hyperglycemia****, i.e. elevated blood glucose****, is common in preterm babies, due to the immaturity of glucose regulation mechanisms; it is often treated with insulin infusion, based on weak evidence.*
- *A number of studies, with heterogeneity in both setting and design, have suggested that hyperglycemia may be associated with increased risk of morbidity in premature infants, especially severe retinopathy of prematurity,* ***a condition characterized by an abnormal development of the retinal vessels that can lead to blindness****.*

b.	Line 92: Please clarify the following a bit more: “…representing a large variation of practices,..”.

*We added to the sentence:*

*“...representing a large variation of practices* ***regarding neonatal management****,...”*

c.	Lines 95-96: Please change “…our results establish that…” to something like “…our results suggest that…”.

*We changed the sentence to “...our results support the hypothesis…”*

d.	Line 106: Please clarify that “…when determining risk of retinopathy.”

*We clarified the sentence as requested:*

*“The results suggest that overall average exposure and duration of hyperglycemia matter more than a single high glucose value* ***when determining risk of retinopathy****.”*

e.	Line 108: Please replace “neonatologists” with “physicians” for clarity.

*We made the change as requested.*

6.	Methods:

a.	Please include day and month in cohort recruitment dates.

*We added day and month in cohort recruitment dates.*

b.	Please clarify whether data were anonymised at time of access, or whether participants provided written informed consent for the use of their data.

*Data were anonymised at time of access; this was clarified in the methods section.*

c.	Please include the relevant prospective analysis plan or protocol with your revised manuscript as a Supporting Information file to be published alongside your study, and cite it in the Methods section, around line 214. A legend for this file should be included at the end of your manuscript.

*We added a Supporting Information file (****S1 Text****. Methods. Preplanned analysis.) and included a reference to it in the Methods section as well as at the end of the manuscript along with other Supporting Information files legends.*

7.	Please report exact p values over 0.001, or p<0.001 otherwise.

*We replaced p values=0.001 with p<0.001 where appropriate as requested.*

8.	At various points (e.g., lines 63, 370) you mention the "large" number of participants. Please clarify that this is large relative to similar studies in the past.

*We made the change as requested.*

9.	Lines 370 and 390: Please replace “prospective” with “prospectively-recruited”, or remove altogether – your analyses are nevertheless retrospective.

*We replaced “prospective” with “prospectively-recruited”.*

10.	PLOS does not permit "data not shown.” Please remove this claim, or do one of the following:
a) If you are the owner of the data relevant to this claim, please provide the data in accordance with the PLOS data policy, and update your Data Availability Statement as needed.
b) If the data not shown refer to a study from another group that has not been published, please cite personal communication in your manuscript text (it should not be included in the reference section). Please provide the name of the individual, the affiliation, and date of communication. The individual must provide PLOS Medicine written permission to be named for this purpose.
c) For any other circumstance, please contact the journal office ASAP.

*We removed this claim.*

11.	Line 423: Please rephrase “consensual” to something like: “Neonatologists are largely in agreement…”

*We rephrased the sentence as follows:*

*“While neonatologists are largely in agreement on the need to treat neonatal hyperglycemia in premature infants...”*

12.	Reference 31: Please check the DOI for accuracy.

*We corrected the DOI from doi:0.1001/jama.300.1.60. to doi:10.1001/jama.300.1.60.*

13.	Please rename the S1 Appendix to “S1 Checklist”.

*We made the change as requested.*

---

Comments from Reviewers:

Reviewer #1: the authors addressed my concerns; I don't have any other issues with this manuscript. 

*We thank Reviewer 1.*

Reviewer #2: The authors have addressed my concerns and I know recommend publication

Peter Flom

*We thank Reviewer 2.*

Reviewer #3: I appreciate the answers to our questions.

*We thank Reviewer 3.*

1. You state in the results that 12.9% of infants in the original cohort had a MaxGly1-21 higher than 20 mmol/L. This results in 111 infants with markedly elevated glucose levels.  This is a very high number of infants with such elevated glucose levels. Was this high a percentage of markedly elevated glucose levels present in the other studies?

*Such data is not available from earlier studies of the association between hyperglycemia and ROP. Most of them studied hyperglycemia as a categorical variable and set the threshold at 8.5 mmol/L or 150 mg/dL. Details of the different glucose levels were not reported. In these studies, 19.4 to 88% of infants experienced a glucose level > 8.5 mmol/L or 150 mg/dL.*

*However, data from the RCT by Bearsdall et al., in which glucose levels of 188 VLBW infants (<1500 g) were prospectively collected during the 1^st^ week of life using continuous glucose monitoring show similar rates of markedly elevated glucose levels compared to our study. In their study, Bearsdall et al. defined as uncontrolled hyperglycemia any episode >20 mmol/L. They observed that uncontrolled hyperglycemia was present in as many as 9% of infants, which is in the range of 12.9% considering our larger period of data collection of 3 weeks.*

2. I agree with you decision to focus on infants with >stage 2 ROP with respect to reviewers 2 comment.

*We thank you.*

3. Table 1 states that the incidence of severe BPD was 6.1% in the primary cohort, but the mean duration of oxygen therapy was 0.4.  Did you look at the infants who developed severe ROP and their duration of oxygen therapy or duration of mechanical ventilation?  Did any infants undergo surgery as that has been recognized as a risk factor for the treatment of severe ROP?

*Duration of oxygen therapy and duration of mechanical ventilation were highly correlated and the association between MaxGly and MeanMaxGly and severe ROP remained unchanged when replacing duration of oxygen therapy with duration of mechanical ventilation in the models.*

*In the primary cohort, 105 (12%) infants underwent surgery. Surgery was indeed associated with severe ROP before and after adjustment for MaxGly (OR 2.28 [95% CI, 1.45-3.51] but this association did not remain significant after adjustment with other confounders (gestational age, birth weight z-score, postnatal weight gain, duration of oxygen supplementation, C-reactive protein or PCT), with an aOR of 0.91 [95% CI, 0.49-1.69]. Because the introduction of the variable “surgery” in the multiple regression analyses did not modify the association between MaxGly or MeanMaxGly and severe ROP, we chose not to amend the manuscript.*

3. You state that the eye exams started at 31 weeks corrected gestation.  It is recommended that these screens start at 31 weeks corrected gestation.  Thus, infants born at 24 weeks are only 28 weeks corrected at 4 weeks of age - well before the timing of identification of ROP. Please discuss why you stopped at 21 days.

*As indicated in the manuscript, clinical signs of ROP are not observed before 4 weeks' age in 99% of infants. To take into account this sequence of events, we included in the analysis the values of glycemia that were measured before ROP was diagnosed only.*

*We agree that babies < 27 WG are less than 4 weeks of age at first screen, but our cohorts also include more mature babies who were only 4 weeks of age at first screen.*

*We chose to stop at 21 days to ensure that all hyperglycemic episodes included in the analyses occurred at least one week before the timing of identification of ROP.*

*In addition, hyperglycemic episodes occur more frequently in the first week of life in premature babies, suggesting that extending the period of data collection to 4 weeks would not make a significant difference.*

*Indeed, in the primary cohort, MaxGly was observed at a mean postnatal age of 5.9 +/- 5.3 days, with a 75th percentile at 9 days. As shown on the graph below, the vast majority of hyperglycemic episodes occurred in the first 3 weeks of life.*

***Figure****: frequencies of post-natal age at which the maximum value of glycemia was observed during the first 28 days in infants in the primary cohort.*

4.Did you describe how many infants in the EPIPAGE cohort received insulin?

*410 of 1441 infants in the EPIPAGE-2 cohort received insulin. The data are indicated in Table S5 (Overall cohort, Non-exposed, n=1031 and Exposed n=410). For more clarity, we added this information in Table S4 legend and in the manuscript.*

5.In the result discussion, you state that the number of infants with severe hyperglycemia above one cut-off level was 97.  However, in table 3, 5 infants with severe ROP and 74 infants without ROP had severely elevated glucose levels. Please explain this discrepancy.

*We thank the reviewer for his/her vigilance. The number of infants with severe hyperglycemia above one cut-off level in the validation cohort is indeed 97. Numbers were reported with an error in the last 2 lines of Table 3, column Validation cohort, for which we sincerely apologize. We made the appropriate correction in Table 3.*
